# Characteristics of Seizure and Antiepileptic Drug Utilization in Outpatients With Autoimmune Encephalitis

**DOI:** 10.3389/fneur.2018.01136

**Published:** 2019-01-08

**Authors:** Qi Huang, Meigang Ma, Xing Wei, Yuhan Liao, Hengchang Qi, Yuejuan Wu, Yuan Wu

**Affiliations:** Department of Neurology, First Affiliated Hospital, Guangxi Medical University, Nanning, China

**Keywords:** autoimmune encephalitis, outpatients, seizure remission, antiepileptic drug withdrawal, refractory seizure

## Abstract

Autoimmune encephalitis (AE) is one kind of encephalitis that associates with specific neuronal antigens. Most patients with AE likely suffer from seizures, but data on the characteristics of seizure and antiepileptic drugs (AEDs) utilization in this patient group remains limited. This study aimed to report the clinical status of seizure and AEDs treatment of patients with AE, and to evaluate the relationship between AEDs discontinuation and seizure outcomes. Patients with acute neurological disorders and anti-N-methyl-D-aspartate receptor (NMDAR), γ-aminobutyric acid B receptor (GABA_B_R), leucine-rich glioma inactivated 1, or contactin-associated protein-like 2 (CASPR2) antibodies were included. As patients withdrew from AEDs, they were divided into the early withdrawal (EW, AEDs used ≤3 months) and late withdrawal (LW, AEDs used >3 months) groups. Seizure remission was defined as having no seizures for at least 1 year after the last time when AEDs were administered. Seizure outcomes were assessed on the basis of remission rate. The factors affecting the outcomes were assessed through Spearman analysis. In total, we enrolled 75 patients (39 patients aged <16 years, male/female = 39/36) for follow-up, which included 67 patients with anti-NMDAR encephalitis, 4 patients with anti-GABA_B_R encephalitis, 2 patients with anti-voltage-gated potassium channel encephalitis, and 2 patients with coexisting antibodies. Among the 34 enrolled patients with anti-NMDAR encephalitis who were withdrawn from AEDs, only 5.8% relapse was reported during the 1-year follow-up, with no significant difference in the percentage of relapse between the EW and LW groups (*P* = 0.313). Fifteen patients (an average age of 6.8, 14 patients with anti-NMDAR encephalitis and 1 patient with anti-CASPR2 encephalitis) presented seizure remission without any AEDs. Seventy five percent of patients with anti-GABA_B_R antibodies developed refractory seizure. Other risk factors which contributed to refractory seizure and seizure relapse included status epilepticus (*P* = 0.004) and cortical abnormalities (*P* = 0.028). Given this retrospective data, patients with AE have a high rate of seizure remission, and the long-term use of AEDs may not be necessary to control the seizure. Moreover, seizures in young patients with anti-NMDAR encephalitis presents self-limited. Patients with anti-GABA_B_R antibody, status epilepticus, and cortical abnormalities are more likely to develop refractory seizure or seizure relapse.

## Introduction

Autoimmune encephalitis (AE) is kind of encephalitis which associates with humoral or cellular responses against specific neuronal antigens (1, 2). The clinical characteristics of these patients include seizure, abnormal behavior, speech dysfunction, movement disorders, and autonomic dysfunction (3). With the development of biochemical assays, several antibodies, such as the anti-*N*-methyl-D-aspartate receptor (NMDAR), anti-γ-aminobutyric acid B receptor (GABA_B_R), anti-leucine-rich glioma inactivated 1 (LGI1), and anti-contactin-associated protein-like 2 (CASPR2) antibodies, have emerged as the leading causes of AE. Therapeutic regimens included first-line immunotherapy (steroids, intravenous immunoglobulin, plasmapheresis), and second-line immunotherapy (rituximab, cyclophosphamide). Despite the severity of the disease in acute phase, most patients recover after proper immunotherapy and intensive support (4).

In acute phase, seizure is a highly prevalent symptom, and parts of patients develop status epilepticus (SE) (5–7). Hence, multiple anti-epileptic drugs (AEDs) are often necessary to control the attacks. However, based on the previous studies and data from our center, most patients likely recover completely after adequate immunotherapy, and seizure is rarely reported in the chronic phase (5, 8, 9). Considering the adverse events of AEDs, some patients stop taking AEDs at the early stage. However, an instructional database describing the long-term use of AEDs with AE is lacking.

This retrospective study aimed to report the seizure characteristics and long-term use of AEDs in outpatients with AE. Our secondary goals included assessing the outcomes between patients with early and late AEDs withdrawal, and determining the probable risk factors for seizure relapse and refractory seizures.

## Materials and Methods

### Study Population

This study was conducted in compliance with the ethical standards of Guangxi Medical University. Written consents were obtained from the patients.

The antibodies, including NMDAR, GABA_B_R, LGI1, and CASPR2, were detected in patients' cerebrospinal fluid and serum samples. The anti-NMDAR antibody was detected by specific staining against NMDAR isolated from rat' hippocampus and cerebellum, and positive cell-based assay with HEK293 cells transfected with NR1. Other antibodies were detected using transfected HEK293 cells with the respective target proteins.

Patients with acute neurological disorders of either sex or any age were considered eligible for this study if they presented any positive antibodies from January 2012 to May 2017. The exclusion criteria included (1) patients diagnosed with epilepsy, cerebral infarction, cerebral trauma, cerebral tumor, and other nervous system disease prior to the onset of encephalitis, (2) patients with evidence of infectious encephalitis, for example, viral, bacteria, mycobacterium tuberculosis, or fungal, (3) patients in the acute phase of autoimmune encephalitis and still required hospitalization. Immunotherapies were used in the acute phase (**Figure 3A**). The decisions about the type and duration of immunotherapy were based on the clinical symptoms, curative, and side effects. The available onset medical data (seizure characteristics, AEDs utilization, and electroencephalogram/neuroimaging findings) were recorded. The electroencephalogram and neuroimaging findings were recorded in the acute phase of the disease.

### Definitions

The chronic stage of AE was defined by 3 months after the onset of AE symptoms. SE was defined as continuous seizure activity lasting >5 min or recurrent seizures without regaining consciousness between seizures for >5 min (10). Refractory seizure was defined as an uncontrolled seizure after treatment by more than three standard therapeutic schedules (11). Seizure remission was defined as having no seizure for at least 1 year after the last time when AEDs were used (12). Seizure with focal characteristics was defined as a partial seizure or a patient with seizure and hemiplegia or hemianesthesia.

### Outcome Assessment and Grouping

AEDs utilization and seizure outcomes were assessed through outpatient services and/or telephone interview. AEDs utilization in the chronic stage (time of continuation/withdrawal) and outcomes (refractory, relapse, or remission) data were collected on patients. Before evaluating the outcomes, the patients who discontinued AEDs were observed for at least 1 year after the last AEDs use.

Patients who withdraw AEDs were divided into two groups based on the duration of AEDs use. The early withdrawal (EW) group had AEDs withdrawn within 3 months, and the late withdrawal (LW) group had AEDs withdrawn after 3 months. Outcomes were assessed based on seizure remission rate and the modified Rankin Scale (mRS) (13).

### Statistical Analysis

Data analysis was performed using IBM SPSS Statistics 22. The skewness and kurtosis coefficients were used to evaluate whether the quantitative data fit a normal distribution. Data were regarded as a normal distribution if the skewness and kurtosis coefficients <1. For the data that did not fit this criterion, we used the Mann–Whitney test to evaluate significance. Other data was compared using a *t*-test or a Fisher's exact test. The factors affecting the outcomes were assessed through Spearman analysis.

## Results

**Figure 1:**
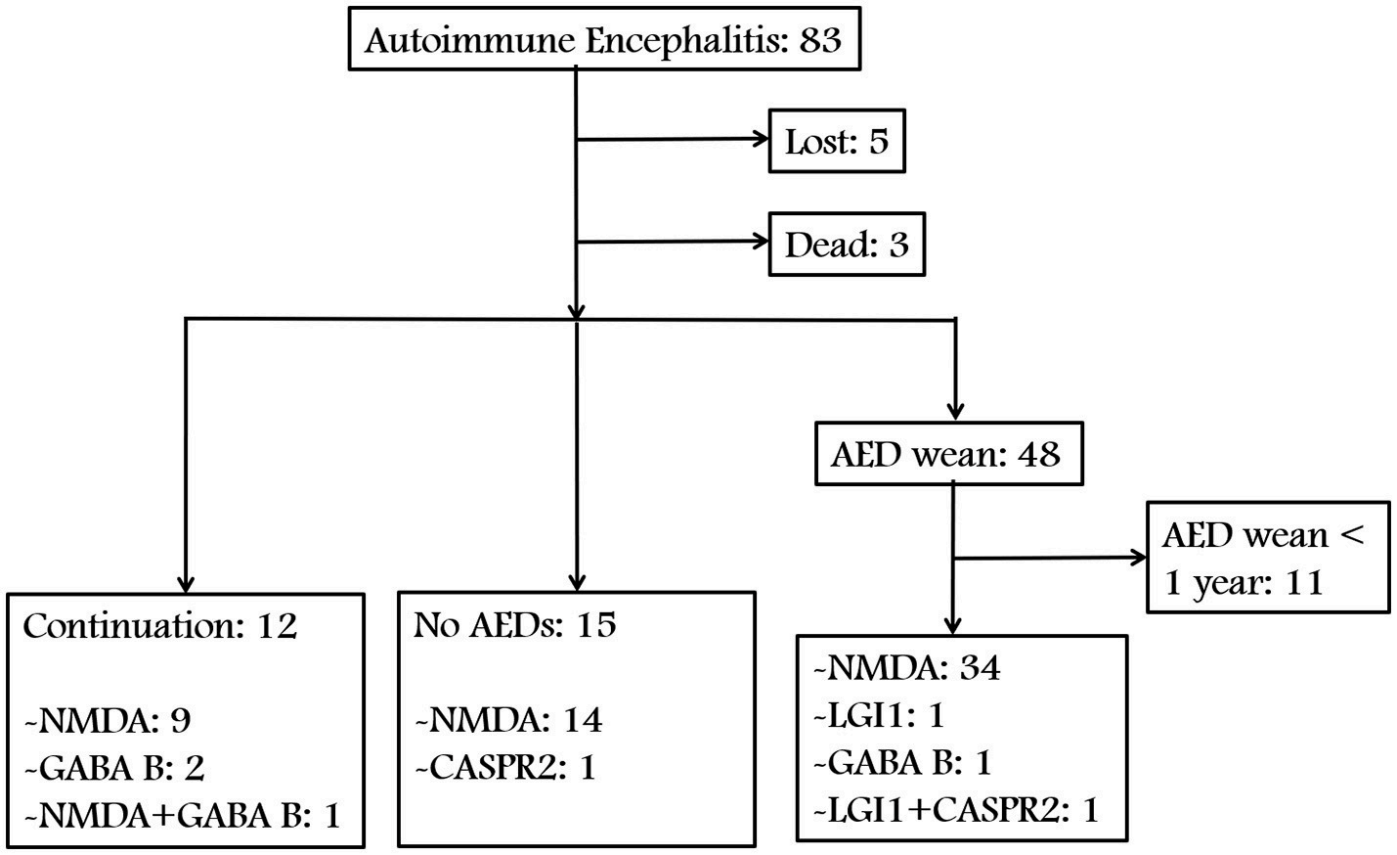
Flow diagram of patient inclusion and grouping.

**Figure 2:**
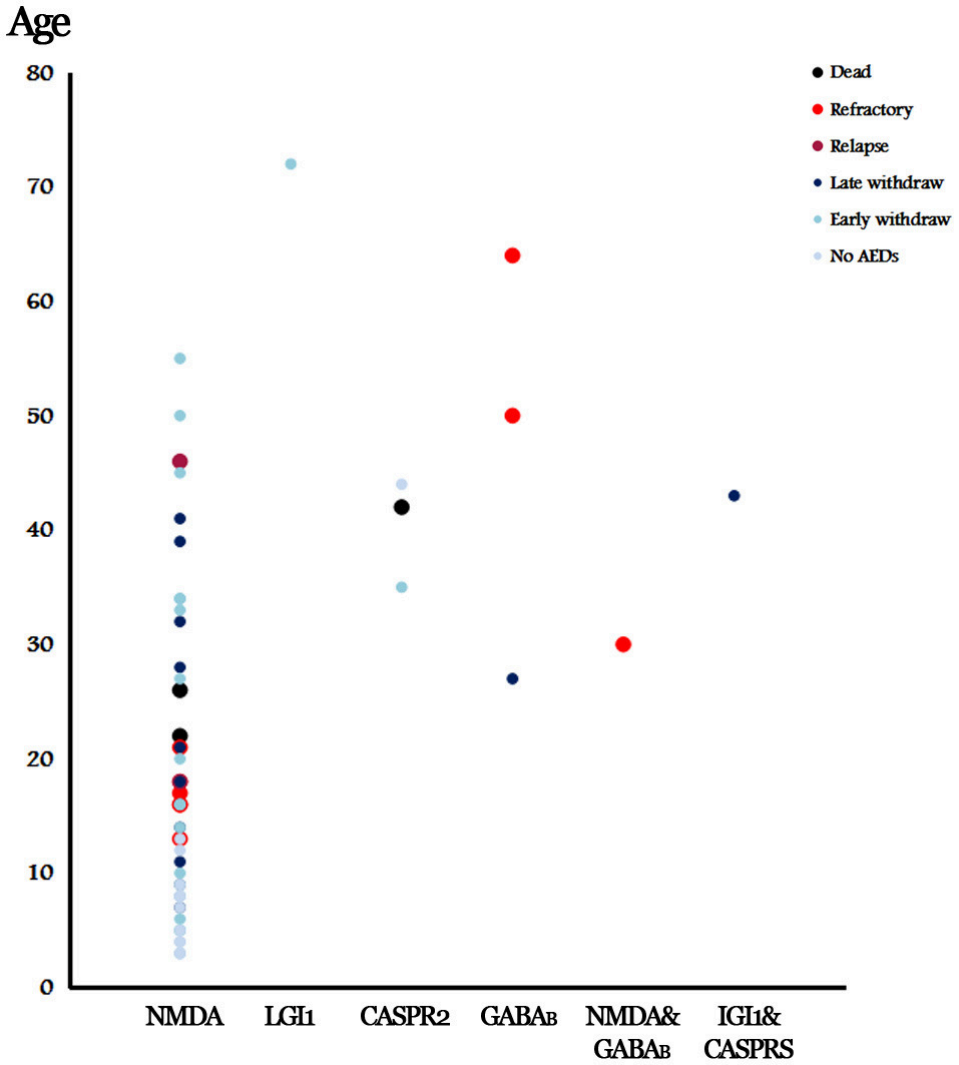
Summary of the presenting patients and outcomes in accordance with the involved antibodies. No AEDs, No AED was given and no relapse was reported. Early withdrawal (EW), AEDs weaned within 3 months and no relapse was reported. Late withdrawal (LW), AEDs weaned after 3 months and no relapse was reported.

We identified 83 patients with AE. Figures 1, 2 show a summary of these patients. In the charts reviewed, 5 patients were lost and 3 patients died of severe pneumonia. As a result, 75 patients were enrolled to follow-up. Among the 75 patients, 12 continued taking AEDs, 15 were not given any AEDs, and 37 patients who withdrew from AEDs were followed up for at least 1 year (Figure 1). The group with AEDs withdrawal included 34 patients with anti-NMDAR encephalitis, 1 with anti-LGI1 encephalitis, 1 with anti-GABA_B_R encephalitis, and 1 patient presented coexisting antibodies of anti-LGI1 and CASPR2 (Figure 1).

### Anti-NMDAR Encephalitis

Among the patients with anti-NMDAR encephalitis, 14 were not given any AEDs, in which 7 suffered from one-time seizure attack at the onset of the encephalitis. Compared to the patients with AEDs, the majority of this group was considerably young (6.8 ± 3.26, *P* = 0.004, Table 1) and less likely to have repetitive seizures (*P* = 0.038, Table 1). The median duration of follow up was 20 months (range: 14–36 months), and no relapse was reported. Furthermore, 12 patients (85.7%) had good outcomes with a 0 mRS score; the remaining 2 patients presented with cognitive dysfunction.

**Table 1:** Clinical characteristics and outcomes of patients with anti-NMDA encephalitis.

	**No AEDs (14 patients)**	**Early withdraw (23 patients)**	**Late withdraw (11 patients)**	**P1**	**P2**
Sex (male/female)	7/7	15/8	5/6	0.474	0.458
Age (Ave ± SD)	6.8 ± 3.26	21.0 ± 16.19	21.4 ± 11.89	0.004	0.935
Seizure frequency				0.038	0.359
None	4	5	2		
Once	7	2	3		
Repeated	3	16	6		
Seizure with focal characters	0	2	0	0.322	0.313
Status epilepticus(Yes/No)	0/14	6/17	5/6	0.024	0.259
Antibody titer				0.129	0.727
1:10	6	3	1		
1:32	7	19	10		
1:100	1	1	0	
MRI abnormalities				0.400	0.329
None	4	6	6		
White matter	3	3	1		
Cortex	3	2	0	
Anti-epileptic drugs				–	0.934
1 kind	–	16	7		
2 kinds	–	5	3		
3 or more kinds	–	2	1		
Followed up					
Median duration (months)	20 (14–36)	36 (15–50)	32 (17–62)	
Relapse	–	2/21	0/11	–	0.313
mRS score (≤1/>1)	12/2	22/1	9/2	0.398	0.239

*AED, Antiepileptic drug. P1, P-value among No AED, Early withdraw, and late withdraw group. P2, P-value between Early withdraw, and late withdraw group*.

**Figure 3:**
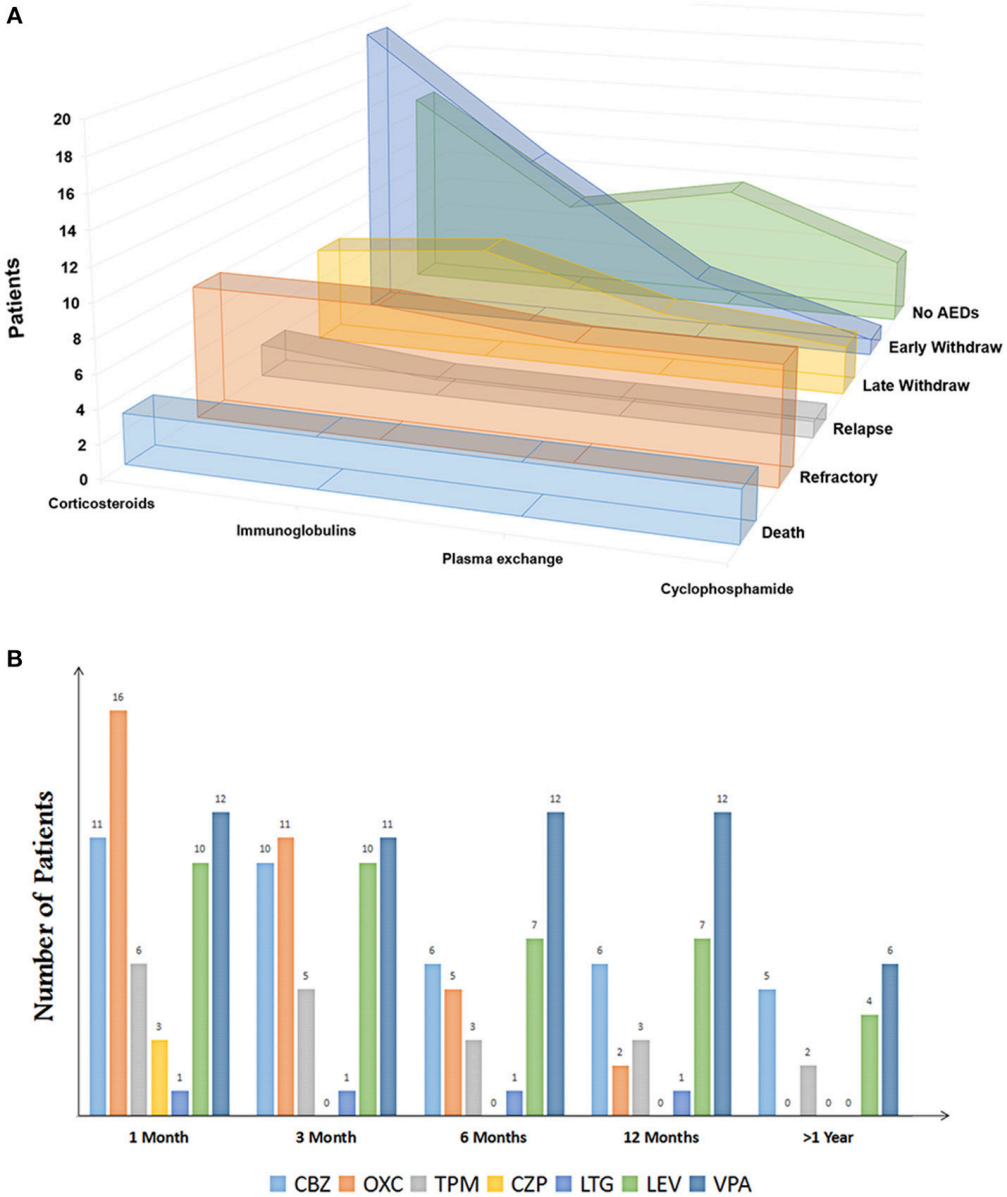
**(A)** Immunotherapies of patients with autoimmune encephalitis (Sort by outcomes). **(B)** Clinical patterns of AED discontinuation of patients with anti-NMDA encephalitis. CBZ, carbamazepine; OXC, oxcarbazepine; TPM, topiramate; CZP, clonazepam; LTG, lamotrigine; LEV, levetiracetam; VPA, valproate.

Among the 34 patients (20 men and 14 women, 23 in EW and 11 in LW) who were discontinued from AEDs, 7 patients (20.6%) did not report seizure, and 5 patients (14.7%) reported a one-time seizure at the onset. Two patients in the EW group reported hemianesthesia before presenting seizures. The occurrence of SE was 32.4% in total. Eighteen patients had an magnetic resonance imaging (MRI) scan and abnormalities were found in 33.3% of the patients, including 4 patients with white matter changes and 2 patients with cortical mass (Table 1). The patterns of AEDs selection and discontinuation were variable (Figure 3B). Monotherapy was the most common selection, with 69.5% in the EW group and 63.9% in the LW group. At the early stage, carbamazepine (*n* = 11) and oxcarbazepine (*n* = 16) were the most chosen AEDs. Valproic acid was among the most commonly continued therapies over the course of follow-up (Figure 3B). Seven patients without seizure were treated with AEDs upon onset. Five of these discontinued AEDs within 1 month, and the remaining 2 patients underwent LW because of frequent subclinical discharge.

The patients' data were compared between the EW and LW groups (Table 1). No statistically significant difference was observed between the two groups in terms of age (*P* = 0.935), sex (*P* = 0.458), seizure characteristics (*P* = 0.359), antibody titers (*P* = 0.727), SE (*P* = 0.259), MRI findings (*P* = 0.329), or AEDs selection (*P* = 0.934). The medium durations of follow up were 36 months (range: 15–50 months) and 32 months (range: 17–62 months) for the EW and LW groups, respectively. 2 patients in the EW group relapsed in the first month after drug discontinuation. No remarkable difference in the percentage of relapse was observed between the two groups.

Detail of patients with anti-NMDAR encephalitis was presented in Supplementary File 1.

### Other AEs

A 44-year-old woman who was diagnosed with anti-CASPR2 encephalitis presented lethargy and headache without seizure at the onset. She did not take any AEDs. No relapse was reported during her 16-month follow up. A 72-year-old man with anti-LGI1 encephalitis and a 35-year-old woman with anti-GABA_B_R encephalitis presented frequent seizures at onset and underwent an AED withdrawal 3 and 6 months later, respectively. No relapse was reported during their 1-year follow up. The other two elder patients (50 and 64 years-old, respectively) with anti-GABA_B_ encephalitis presented refractory seizures (Figure 2).

We also reviewed 2 coexisting AE. One is a 30-year-old female who presented with anti-NMDAR and GABA_B_R encephalitis. She had a seizure 10 years ago before she was diagnosed with AE through CSF detection. Her EEG presented δ brushes with generalized paroxysmal θ activities. The brain MRI was unremarkable. She developed refractory seizures after being treated by oxcarbazepine, carbamazepine, and clonazepam. The other patient was a 43-year-old woman with LGI1 and CASPR2 antibodies. She presented repeated generalized tonic-clonic seizure and paroxysmal speech dysfunction. Bilateral paracentral lesion was found in the MRI scan. She was given levetiracetam and valproic acid. The AEDs were withdrawn 2 years later, and no relapse was reported during her 1-year follow up (Figure 2).

### Risk Factors

Among the 12 patients who remained on AEDs, 9 patients presented refractory seizure, including 6 patients with anti-NMDAR encephalitis, 2 patients with anti-GABA_B_R encephalitis, and 1 patient with anti-NMDAR and GABA_B_R encephalitis (Figure 2). Among patients with anti-NMDAR encephalitis, refractory seizures occurred more often in the patients younger than 30 years of age (Figure 2), and who presented repetitive seizures and SE, moreover, 4 patients (66%) showed cortical abnormalities on the MRI scan.

By combining the data of the 2 relapse cases, we evaluated the risk factors that contributed to the worse outcomes of anti-NMDAR encephalitis. The patients with relapse or refractory seizure were more likely to be accompanied with cortical lesions on MRI (*P* = 0.028) and SE (*P* = 0.004) than those who reached seizure remission (Table 2). Moreover, the seizures with focal characteristics (*P* < 0.001), SE (*P* < 0.001), and MRI abnormalities (*P* = 0.010) were significantly associated with refractory seizure (Table 2). For other kinds of AEs, we found 3 patients with anti-GABA_B_R encephalitis (75% in all, including 1 patient with concomitant disease) developed refractory seizure; this number is much higher than those of others.

**Table 2:** Spearman analysis of factors associated with outcomes.

	**Refractory Seizure/P**	**Relapse/P**	**Both/P**
Sex	0.571	0.859	0.516
Age	0.528	0.116	0.259
Antibody titers	0.640	0.590	0.555
Seizure with focal characters	<0.001	0.769	0.099
Status epilepticus	<0.001	0.415	0.004
MRI abnormalities	0.010	0.961	0.028

## Discussions

Parts of AE have been linked to cell surface antigens, which included voltage-gated potassium channel (VGPC, e.g., LGI1 and CASPR2), NMDAR, and GABA_B_R (14). To evaluate the AEDs utilization associated with AE, we focused on seizure in a cohort of patients with anti-NMDAR, anti-GABA_B_R, anti-LGI1, and CASPR2 encephalitis. This study demonstrated low recurrence rates in young patients with AE who experienced first unprovoked seizures and highlighted an overall remission rate of 94% after the patients discontinued AEDs therapy. No difference was noted between the EW (≤3 months) and LW (>3 months) of AEDs. Moreover, a higher number of patients with anti-GABA_B_R antibody, SE, cortical abnormalities, and focal neurological dysfunction experienced refractory seizure or seizure relapses compared to those who did not.

### Seizure Remission in Anti-NMDAR Encephalitis

Since 2005, the characteristics and long-term outcomes of anti-NMDAR encephalitis have aroused public attention due to the high incidence in young patients with serious neurological dysfunctions (15). According to a previous study, we found that the probable predicted factors for poor outcomes in the acute phase included older age, altered consciousness, and SE, and the process of terminating SE was particularly important for anti-NMDAR encephalitis (5). By evaluating patients through the mRS score, the other multi-institutional study which included clinical data from 577 patients with anti-NMDAR antibodies observed that 81% of the patients responded to immunotherapy (8). Seizure occurs as a prominent feature in AE (16), whether long-term AEDs are necessary after patients achieving good outcomes has not been established (14, 16).

AEDs withdrawal after a successful seizure control may prevent adverse side effects and excessive cost. However, the studies which have evaluated the safety of AEDs weaning in patients with anti-NMDAR encephalitis are rare. One study demonstrated that no difference in seizure recurrence between 1 and 2 years of AEDs therapy in children with viral encephalitis (17), and the relative risk factors might have included EEG abnormalities or HIV infection (18). However, the duration of AEDs in anti-NMDAR encephalitis tended to be shorter (19). Among the 34 enrolled patients with AEDs withdrawal, only 5.8% suffered from relapse during the follow up. Moreover, no difference was found between the EW (≤3 months) and LW (>3 months) groups, which is consistent with that of a previous study (16).

Information regarding AEDs use in adolescents and adults with anti-NMDAR encephalitis has been reported previously, but data from younger children remain limited (16). In the present study, we enrolled 14 young patients with average age of 6.8 (range: 3–12 years). Compared with the older age group, the young group was more likely be seizure free during the long-term follow-up without AEDs, even if they suffered one-time seizure attacks at the onset. The key treatment decisions after the first unprovoked seizure are a controversial issue in children (20, 21). In some epilepsy syndromes, such as in benign childhood epilepsy with centrotemporal spikes or childhood absence epilepsy, remission is a regular feature of the natural history, whereas juvenile myoclonic epilepsy or other symptomatic epilepsies have long been considered to present in AEDs continuation (22). By using the statistical methods for survival analysis, one study indicated that the cumulative risk of repetitive seizure in children was 29% in the first year and elevated to 45% within the 10-year follow up (23). However, the factors associated with recurrences after the first seizure are complex. The probable risk factors may include age (24) and etiology (25). For anti-NMDA encephalitis, our data indicated that seizure in young patients tended to be self-limited. Moreover, some scholars indicate that a cognitive comorbidity likely accompany with the initial seizure if AEDs are not used (26). However, evaluation of mRS score revealed that the majority of children in this group presented normal daily activities.

Although our data supports that seizure remission is common and that long-term use of AEDs may not be necessary, the significance of immunotherapy cannot be ignored to control the seizure. Numerous studies demonstrated that it is the immunotherapies that control the symptoms in anti-NMDAR encephalitis (5, 8, 9). Moreover, one retrospective study which focused on the outcome of AEDs alone in controlling the seizure of patients with anti-VGPC-complex antibodies indicated that only 23.5% of patients became seizure free compared with 61.5% of patients with immunotherapy (14).

### Seizure Remission in Anti-GABABR, LGI1, and CASPR2 Encephalitis

Anti-GABA_B_R, LGI1, and CASPR2 antibodies have been recently detected in patients with limbic encephalitis (27). Seizures are frequently reported in limbic encephalitis, but autonomic and psychiatric symptoms are more highlighted. In these antibody-mediated seizures, remission rate is variable, and may be related to complications, immunotherapy, and ICU management (7).

Although rare, seizure with additional auto-antibodies may be a other probable risk factor. LGI1 and CASPR2 are the extracellular domains of VGPC. The coexistence of anti-LGI1 and anti-CASPR2 encephalitis may contribute to seizure, cognitive disturbance, movement disorders, and pain (7). Besides AEDs use, the empirical approach to seizure control is corticosteroid treatment (28, 29). A GABA_B_R antibody is rarely accompanied with other antibodies in a same patient, and a probable reason may involve different genetic predispositions (30, 31). In our study, the presented patients developed refractory seizure and severe cognitive dysfunction. One possible mechanism of AEDs resistance may be associated with different interaction sites, as anti-NMDAR antibodies have been suggested to decrease the synaptic levels of receptors, whereas the anti-GABA_B_R antibody would further alter the synaptic function (32).

### Risk Factors for Refractory Seizure and Seizure Relapse

As AEDs are supposedly unnecessary to seizure outcomes in AE after appropriate immunotherapy, we evaluate the other probable independent risk factors that contribute to refractory seizure and seizure relapse. In addition to involving the anti-GABA_B_R antibody, our result shows that patients with SE, cortical abnormalities in MRI, and focal neurological dysfunction are more likely to develop worse outcomes than those who did not accompany these findings. SE has been proven to be an independent risk factor in major types of epilepsy (33, 34). The complications of SE, such as severe pneumonia and ICU admission, have also contributed to poor outcomes (5). Cortical abnormalities seem positively correlate with refractory seizure. By using structural MRI scans, one study demonstrated a strong association between incomplete recovery and superficial white matter lesions (35). This observation indicates that the injured connection between adjacent cortices may play a crucial role in seizure control after AE.

## Limitations

This study has some limitations. First, we tried to discuss the risk factors of seizure outcomes for AE after appropriate immunotherapy. However, as antibodies were not measured at later time points, the direct relationship between persistent antibody levels and seizure outcomes remained unclear. A previous study presented that the neurological recovery accompanied with reduced antibody titer (15), while the correlation between antibody titer and refractory seizure showed unremarkable in our study. One probability which caused the difference might involve the time when antibody was measured. Though the seizure outcomes were assessed based on the data of outpatients, the patients who developed refractory seizure might have persistent higher titer level than those who did not. Second, as the relapse rate was altered with the follow-up period (23), long follow-up times are necessary. However, 54–100% of seizures after AE occurred within the first year, and new-onset seizure after 1 year of follow up was rare (36–38). Third, the results were limited because of the relatively small cohort. Combined with the previous data (16), a probable correlation between seizure remission and AED utilization was noted, and further trials and meta-analysis are needed to confirm these results.

## Conclusion

Patients with AE have a high rate of seizure remission after proper immunotherapies. The long-term use of AEDs appears not be necessary to control their seizures. Compared with adults, young patients are more likely to become seizure free without AEDs. The risk factors that contribute to refractory seizure or seizure relapse may include anti-GABA_B_R encephalitis, SE, and cortical abnormalities.

## Author Contributions

QH is the first author of this manuscript. YuaW is the corresponding author of this manuscript, they contributed to the study design, data collection and analysis, and draft writing of this work. MM, XW, and YL have revised and improved the manuscript. HQ and YueW contributed in the data collection and figures. All authors have seen and agreed on the finally submitted version of the manuscript.

### Conflict of Interest Statement

The authors declare that the research was conducted in the absence of any commercial or financial relationships that could be construed as a potential conflict of interest.
