# Supplementary material for: Characteristics of Seizure and Antiepileptic Drug Utilization in Outpatients With Autoimmune Encephalitis
Source: Front Neurol. 2019 Jan 8;9:1136. doi: 10.3389/fneur.2018.01136 (PMC6331521; doi:10.3389/fneur.2018.01136)
Supplement: Supplementary file 1 [file Table_1.docx]

| **Supplementary file 1** The details of patients with anti-NMDA encephalitis | | | | | | | | | | |
| --- | --- | --- | --- | --- | --- | --- | --- | --- | --- | --- |
|  | | | | | | | | | | |
| **Patients’**  **No.** | **Sex** | **Age** | **Seizure Frequency** | **SE** | **Antibody Titer** | **MRI** | **AEDs** | **Follow-up** | **AED wean** | **Outcomes** |
| 51 | Male | 5 | Repeated | No | 1:32 | Cortex | CZP, VPA | 18 | Early Withdraw | Remission |
| 1 | Male | 11 | Once | No | 1:32 | - | TPM | 36 | Late withdraw | Remission |
| 33 | Female | 16 | Repeated | Yes | 1:32 | - | CBZ, TPM, LEV | 18 | Late withdraw | Remission |
| 52 | Male | 8 | - | No | 1:32 | White Matter | - | 18 | No AEDs | Remission |
| 43 | Male | 34 | Repeated | Yes | 1:32 | Cortex | OXC, TPM | 50 | Early Withdraw | Remission |
| 36 | Male | 45 | - | No | 1:32 | White Matter | OXC | 16 | Early Withdraw | Remission |
| 2 | Male | 22 | Repeated | Yes | 1:32 | Normal | VPA, LEV | - | - | Dead |
| 53 | Female | 4 | Once | No | 1:10 | Cortex | - | 18 | No AEDs | Remission |
| 3 | Male | 3 | - | No | 1:10 | - | OXC | 36 | Early Withdraw | Remission |
| 4 | Male | 18 | Repeated | Yes | 1:10 | Normal | CBZ, TPM | 32 | Late withdraw | Remission |
| 54 | Male | 8 | Repeated | No | 1:10 | - | - | 16 | No AEDs | Remission |
| 55 | Female | 8 | Repeated | No | 1:100 | White Matter | - | 16 | No AEDs | Remission |
| 56 | Female | 8 | Once | No | 1:100 | - | CZP | 26 | Early Withdraw | Remission |
| 5 | Female | 8 | Repeated | Yes | 1:100 | Cortex&White Matter | CBZ, VPA, TPM | 43 | Continuation | Refractory |
| 6 | Female | 34 | Repeated | Yes | 1:32 | Normal | OXC | 41 | Early Withdraw | Remission |
| 57 | Female | 3 | Once | No | 1:32 | Normal | - | 22 | No AEDs | Remission |
| 27 | Male | 28 | - | No | 1:32 | Normal | OXC | 28 | Late withdraw | Remission |
| 42 | Male | 17 | Repeated | Yes | 1:32 | - | VPA, OXC | 12 | Continuation | Control |
| 7 | Female | 32 | Repeated | Yes | 1:32 | Normal | TPM | 34 | Late withdraw | Remission |
| 8 | Female | 10 | Repeated | No | 1:32 | - | OXC | 37 | Early Withdraw | Remission |
| 44 | Female | 21 | Once | No | 1:32 | Normal | LTG, LEV | 62 | Late withdraw | Remission |
| 9 | Female | 18 | Repeated | No | 1:32 | White Matter | LEV, OXC | 46 | Early Withdraw | Relapse |
| 10 | Female | 9 | Once | No | 1:32 | Normal | OXC | 39 | Late withdraw | Remission |
| 11 | Male | 8 | Repeated | No | 1:32 | - | TPM | 46 | Early Withdraw | Remission |
| 12 | Female | 26 | Repeated | Yes | 1:32 | Cortex | LEV, OXC | - | - | Dead |
| 31 | Female | 20 | - | No | 1:32 | Normal | CBZ | 26 | Early Withdraw | Remission |
| 13 | Female | 9 | Repeated | No | 1:32 | - | - | 36 | No AEDs | Remission |
| 35 | Male | 3 | Repeated | No | 1:10 | Cortex | - | 14 | No AEDs | Remission |
| 14 | Female | 13 | Once | No | 1:10 | White Matter | - | 34 | No AEDs | Remission |
| 38 | Female | 3 | Repeated | Yes | 1:10 | Cortex | LEV, TPM, OXC, LTG | 22 | Continuation | Refractory |
| 41 | Male | 14 | - | No | 1:10 | - | CBZ | 14 | Early Withdraw | Remission |
| 34 | Male | 8 | - | No | 1:32 | Normal | - | 36 | No AEDs | Remission |
| 45 | Male | 16 | Repeated | Yes | 1:10 | - | LEV, VPA, LTG | 12 | Continuation | Refractory |
| 46 | Male | 16 | Repeated | No | 1:10 | - | OXC | 40 | Early Withdraw | Remission |
| 15 | Male | 6 | Repeated | Yes | 1:32 | - | CBZ, VPA, TPM | 38 | Early Withdraw | Remission |
| 16 | Male | 12 | - | No | 1:10 | - | - | 33 | No AEDs | Remission |
| 59 | Male | 7 | Once | No | 1:32 | Normal | - | 16 | No AEDs | Remission |
| 28 | Male | 27 | Repeated | No | 1:32 | - | VPA, CBZ | 28 | Early Withdraw | Remission |
| 47 | Female | 16 | Repeated | Yes | 1:32 | Cortex | VPA, CBZ, TPM | 72 | Continuation | Refractory |
| 17 | Male | 4 | - | No | 1:32 | Normal | - | 36 | No AEDs | Remission |
| 18 | Female | 5 | Repeated | No | 1:32 | Normal | OXC | 38 | Early Withdraw | Remission |
| 19 | Female | 5 | Repeated | Yes | 1:32 | Cortex | - | 36 | No AEDs | Remission |
| 60 | Male | 7 | Repeated | No | 1:32 | Normal | VPA | 14 | Continuation | Control |
| 32 | Female | 41 | - | No | 1:32 | - | OXC | 17 | Late withdraw | Remission |
| 20 | Male | 39 | Repeated | Yes | 1:32 | White Matter | CBZ, VPA | 27 | Late withdraw | Remission |
| 21 | Male | 46 | Repeated | No | 1:32 | Normal | CBZ | 37 | Early Withdraw | Relapse |
| 22 | Male | 16 | Repeated | Yes | 1:32 | Normal | OXC | 31 | Early Withdraw | Remission |
| 23 | Male | 5 | Once | No | 1:32 | - | CBZ | 36 | Early Withdraw | Remission |
| 61 | Male | 9 | Repeated | No | 1:32 | Normal | CZP | 41 | Early Withdraw | Remission |
| 39 | Female | 50 | - | No | 1:32 | White Matter | OXC | 15 | Early Withdraw | Remission |
| 24 | Male | 7 | Repeated | Yes | 1:32 | - | VPA | 36 | Late withdraw | Remission |
| 25 | Female | 21 | Repeated | Yes | 1:32 | Cortex&White Matter | LEV, VPA | 64 | Continuation | Control |
| 29 | Female | 13 | Repeated | Yes | 1:32 | Cortex | CBZ, CZP, VPA | 26 | Continuation | Refractory |
| 48 | Male | 18 | Repeated | No | 1:32 | - | VPA, LEV, TPM | 12 | Continuation | Refractory |
| 37 | Female | 3 | Once | No | 1:10 | - | - | 14 | No AEDs | Remission |
| 49 | Female | 55 | Repeated | Yes | 1:32 | - | OXC, LEV | 12 | Early Withdraw | Remission |
| 30 | Male | 33 | Repeated | No | 1:32 | - | OXC | 25 | Early Withdraw | Remission |
| 26 | Male | 16 | Repeated | Yes | 1:32 | - | LEV, OXC, TPM | 33 | Early Withdraw | Remission |
| 62 | Female | 14 | Repeated | No | 1:32 | Normal | OXC | 18 | Late withdraw | Remission |

SE: Status epilepticus; CBZ: carbamazepine; OXC: oxcarbazepine; TPM: topiramate; CZP: clonazepam; LTG: lamotrigine; LEV: levetiracetam; VPA, valproate.
